# Naturally Occurring Food Toxins

**DOI:** 10.3390/toxins2092289

**Published:** 2010-09-20

**Authors:** Laurie C. Dolan, Ray A. Matulka, George A. Burdock

**Affiliations:** Burdock Group, 801 N. Orange Ave., Suite 710, Orlando FL 32801, USA; Email: rmatulka@burdockgroup.com (R.A.M.); gburdock@burdockgroup.com (G.A.B.)

**Keywords:** toxin, natural, environmental, exposure, processing, cooking, food

## Abstract

Although many foods contain toxins as a naturally-occurring constituent or, are formed as the result of handling or processing, the incidence of adverse reactions to food is relatively low. The low incidence of adverse effects is the result of some pragmatic solutions by the US Food and Drug Administration (FDA) and other regulatory agencies through the creative use of specifications, action levels, tolerances, warning labels and prohibitions. Manufacturers have also played a role by setting limits on certain substances and developing mitigation procedures for process-induced toxins. Regardless of measures taken by regulators and food producers to protect consumers from natural food toxins, consumption of small levels of these materials is unavoidable. Although the risk for toxicity due to consumption of food toxins is fairly low, there is always the possibility of toxicity due to contamination, overconsumption, allergy or an unpredictable idiosyncratic response. The purpose of this review is to provide a toxicological and regulatory overview of some of the toxins present in some commonly consumed foods, and where possible, discuss the steps that have been taken to reduce consumer exposure, many of which are possible because of the unique process of food regulation in the United States.

## 1. Introduction

Historically, we have learned that everything is toxic; it is only the dose that separates the toxic from the non-toxic. Even water is toxic if a large amount (4–5 liters) is consumed in a relatively short time (2–3 hours). The pathogenesis of water intoxication includes hyponatremia, followed by cerebral edema, seizures and death.

Like water, too much of a good thing such as the antioxidant vitamin A, can have acute toxic effects leading to hepatotoxicity [1] or chronic high levels can have a pro-oxidant effect [2]. Something as innocent as licorice, when consumed in large amounts may be harmful. For example, Bannister and associates reported hypokalemia leading to cardiac arrest in a 58-year-old woman who had been eating about 1.8 kg of licorice per week [3]. This licorice intoxication (dubbed “glycyrrhizism” after glycyrrhizic acid, the active component of licorice), has an effect resembling that of aldosterone, which suppresses the renin-angiotensin-aldosterone axis, resulting in the loss of potassium. Clinically, hypokalemia with alkalosis, cardiac arrhythmias, muscular symptoms together with sodium retention and edema, and severe hypertension are observed. The syndrome may develop at a level of 100 g licorice per day but gradually abates upon withdrawal of the licorice [4].

Recently, public health and social agendas have become more proactive in food toxicology, such as regulating (or outright banning) trans fats or “endocrine disruptors” in foods on the basis of public safety, including a suggestion of removing the generally recognized as safe (GRAS) status for salt [5]. These agendas lose sight of the basic principle of toxicology that “the dose makes the poison” and that demanding “safety *per se*” or “safe at any dose”, for all foods and ingredients is a non-starter and as a concept, was abandoned with the adoption of the Federal Food and Drug Act (FFDCA) in 1958. For their part, the regulators can limit amounts of potentially toxic substances allowed in food and in those circumstances where setting limits is not effective and public health policy makers provide the public with sufficient information (e.g., label information), where possible, to protect the consumer from reasonably foreseeable problems. Labeling requirements by the FDA provide the consumer with helpful information about content of fats, carbohydrate, protein, potential allergens, caloric value, *etc.*, but do not provide information about toxins that may be inherent in the foods or formed during processing. Because some food toxins cannot be removed from foods and others may be created during processing or cooking, consumption of small quantities of food toxins is unavoidable. The purpose of this review is to illustrate the potential risks of these toxins when consumed at concentrations normally present in foods and the steps taken by regulators to mitigate exposure where possible. Although regulatory information from countries other than the United States is included, FDA legislation is emphasized. Readers from other countries are advised to consult regulations for their specific region, because regulations and regulatory practices in other countries may differ from those in the United States.

## 2. Regulatory Accommodation

Foods are regarded as such because they are edible—they cannot be unpalatable or toxic—and; foods must have nutritional, hedonic or satietal value—otherwise there would be no point in consuming them. Therefore, in the absence of a spontaneous change or contamination, the concept of a toxic food *per se* would seem to be an oxymoron. How then, could a food be toxic and still be considered a food—there are two principal means: (1) an ordinarily non-toxic food has become toxic, if even for a small subpopulation; and (2) over-consumption of an ordinarily non-toxic food. This shift between toxic and non-toxic or toxic only for a select group has the potential for creating headaches for regulatory agencies charged with protecting the health of the public, but as the reader will see in the following pages, the FDA and other regulatory agencies have created some thoughtful and pragmatic solutions for achieving a balance of acceptable risk and unavoidable circumstances.

The large diversity of acceptable foods made it difficult for the framers of the Federal Food Drug and Cosmetic Act (FFDCA) to define what a food could be, so they settled on the pragmatic definition provided in §201(f) [6]:

The term “food” means (1) articles used for food or drink for man or other animals, (2) chewing gum, and (3) articles used for components of any such article.

The framers are to be congratulated on their realistic approach, but a little interpretation is required. In the first clause “…articles used for food...” includes what humans and animals will eat as such (including eggs, meat, kohlrabi, Velveeta^®^ cheese and angel food cake). The third clause “articles used for components of any such article,” are simply those substances used to make food (defined in the first clause)—therefore, anything approved for addition to food, becomes a part of the food. The second clause was more of a political consideration than anything else, as there was some disagreement whether chewing gum was swallowed or expectorated; the swallowers prevailed and chewing gum is regulated as a food. Had the majority determined that chewing gum was expectorated (as is evident on a sidewalk outside of any theater or church), it would have been classified with breath mints (which are not swallowed) and are therefore regulated as a cosmetic, whose function is to “…promote attractiveness…” of the body [6]. It has also been ruled by the FDA that proposed dietary supplements (which are regulated as a subset of foods) meant to be held in the mouth, followed by expectoration, are not dietary supplements, because they are not swallowed.

The definition of food has generally held since the 1958 definition, although it was changed slightly in the 7th Circuit in 1983, to now indicate that a food is something consumed “…primarily, for [it’s] taste, aroma or nutritive value.” This court decision did not radically change the definition of food from the original context, but in this particular case, prohibited the use of a food extract for therapeutic intent (*i.e.*, amylase isolated from kidney beans as an inhibitor of carbohydrate breakdown and marketed for weight loss—so-called “starch blockers”).

In general, the law prohibits the sale of food “if it consists in whole or in part of any filthy, putrid, or decomposed substance, or if it is otherwise unfit for food” (in practice, “fitness” can be quite subjective). Also, some foods which are ordinarily safe to eat may become unsafe, as described in §402 of the FFDCA [7]:

§402. A food shall be deemed to be adulterated—(a) (1) If it bears or contains any poisonous or deleterious substance which may render it injurious to health; but in case the substance is not an added substance such food shall not be considered adulterated under this clause if the quantity of such substance in such food does not ordinarily render it injurious to health…

The first part of §402 is clear; if a food contains a poisonous or deleterious substance it cannot be used as a food—a fairly broad standard. The second part of the section “…but in case the substance is not an added…the quantity of such substance does not ordinarily render it injurious to health…” requires an explanation. This clause simply means that although toxic substances may be present in foods, the food is not adulterated if the amount present in the food is not ordinarily injurious to health. For example, tomatine in tomatoes, psoralens in celery or glycoalkaloids in potatoes are normally present in concentrations that are not harmful; however, in the event these amounts are increased (through such processes as breeding, mishandling during harvesting, storage or transportation) and become harmful, these foods are then considered to be adulterated. This second and narrower part of the statute is followed up in §406 of the FFDCA [8]:

§406 Any poisonous or deleterious substance added to any food, except where such substance is required in the production thereof or cannot be avoided by good manufacturing practice shall be deemed to be unsafe for purposes of the application of clause (2) (A) of section 402(a); but when such substance is so required or cannot be so avoided, the Secretary shall promulgate regulations limiting the quantity therein or thereon to such extent as he finds necessary for the protection of public health, and any quantity exceeding the limits so fixed shall also be deemed to be unsafe for purposes of the application of clause (2) (A) of section 402(a).

§406 then, allows the FDA to establish tolerances for these unavoidable contaminants, that is, a food may contain a toxin (such as mercury), if the presence of that toxin is (a) unavoidable and (b) under the level tolerated, the food is not considered to be unsafe. Because establishing a “tolerance” requires an extensive rule-making process, the FDA has adopted the use of “action levels”, which are non-binding guidelines [9]. For food ingredients (e.g., additives), potentially harmful constituents or contaminants are addressed by limiting the amount present in the specifications; higher than allowed amounts render the ingredient and the food to which it has been added, adulterated.

A few potential foods are banned outright by regulation such as the slaughter of companion animals (cats, dogs and horses) for food, offal and colostrum or those foods whose preparation is regulated by guidelines other than current good manufacturing practices (e.g., pufferfish preparation). Some naturally sourced substances (while present in some foods) are banned for addition to food for reasons of safety and include safrole, calamus and coumarin (a full list of which may be seen in 21 Code of Federal Regulations (CFR) 189). Other foods which may contain toxic substances, such as prussic acid in peach leaves, β-thujone in wormwood, saxitoxin in seafood, *etc.*, are controlled by regulation through the use of tolerances, or more correctly, specifications for the product that limit the amount of toxin that may be present. For those foods or ingredients with potential for harm, but not addressed by a specific regulation, action level, *etc.*, the reference in the FFDCA to substances “unfit for food” and flowing from that provision, Sections 402 and 406 of the FFDCA, apply. That is, the lack of a specific action taken by the FDA (or any regulatory agency), for a potentially harmful substance is not a license to market that substance.

## 3. Factors Driving the Acceptance of Certain Foods

Beyond the basic requirements of nutritional or hedonic value, the concept of exactly what constitutes food is largely culturally based; that is, the consumption of pork, shellfish, eel, “rocky mountain oysters”, cracklings, chitlin’s (chitterlings), brain, monkey, guinea pig, dog, snake, insects and arachnids, *etc.*, may be prohibited by religious practices or a matter of personal taste and, in the case of brains (or neural tissue) at least from cattle, has recently become no longer acceptable. Interestingly, there are no fruits or vegetables on any theocratic forbidden list. 

There are some personal prohibitions that are genetically driven, but may not be perceived as a “toxicity” concern. For example, a genetic variant has been described for cilantro, which is perceived by some people as having an unpleasant soapy taste or rank smell [10]. Another, better known variant is the ability to taste phenylthiourea (also known as phenylthiocarbamide, PTU or PTC) [11]. The ability to taste and smell certain substances may be key to evolutionary survival, as while the alkaloids of many potentially poisonous plants confer a bitter flavor, Goff and Klee have indicated that certain flavors and odors may also provide sensory cues for nutritional value of some plants [12]. For example, the characteristic odor profile of tomato (e.g., “tomato”, “green”, or “grassy”) are derived from *cis*-3-hexenal, *cis*-3-hexenol and *trans-*hexenal along with visual cues, to promote repeated consumption of an enjoyable food. In the context of promoting consumption of a specific food anosmia (lack of odor perception) or “specific anosmia” (which may be genetically based), will put the individual at a competitive disadvantage in food selection. Persistent or total anosmia also represents a clear safety hazard as the individual could not detect the tell-tale signs of decay or putrefaction of unfit foods.

There are some food prohibitions that are medically driven, as the result of genetics or autoimmune disease, as shown in Table 1.

**Table 1:** Medically driven food prohibitions (compiled from NORD [13]).

Disease/Syndrome	Causative Food	Cause	Comment
Disaccharide intolerance	Sucrose, dextrins	Autosomal recessive trait characterized by the deficiency or absence of enzymes sucrase and isomaltase in the intestine.	Attacks characterized by bloating and diarrhea.
Favism	Broadbean (*Vicia fava*)	X-linked recessive trait resulting in low amounts of glucose-P-dehydrogenase. Several subtypes known.	Hemolytic anemia may result from consumption of offending foods.
Galactosemia	Galactose and lactose (dairy products)	Autosomal recessive trait with low levels of any one of three enzymes directly responsible for galactose metabolism.	High levels of galactose in the blood results in hepatomegaly, cirrhosis, and renal failure. Infant mortality is ~75%.
Gluten intolerance	Wheat, barley, gluten containing foods	Autoimmune disease	Sensitivity to storage protein (gliadin) in some grains.
Lactose intolerance	Dairy products	Inborn error of metabolism—low or no lactase enzyme in the intestine.	Lactase is required to cleave lactose (a disaccharide of galactose and glucose). Bloating and diarrhea may develop.
Ornithine transcarbamylase deficiency	Dietary nitrogen (primarily meat)	X-linked recessive disorder resulting in low production of hepatic ornithine transcarbamylase interrupting the urea cycle and leading to accumulation of ammonia.	Although usually first seen in neonates, there may be an adult onset.
			Citrullinemia is another genetic disease affecting the urea cycle.
Phenylketonuria (PKU disease)	Phenylalanine in foods	Autosomal recessive trait characterized by inadequate hepatic phenylalanine hydroxylase.	Leads to accumulation of phenylpyruvate which may accumulate in the brain and lead to seizures, mental retardation, *etc.* Products containing phenylalaine must be labeled.
Refractory sprue	Wheat, barley and rye	Autoimmune disorder triggered by gliadin, a gluten storage protein.	Unlike common celiac sprue, adherence to a gluten-free diet may not cause symptoms to abate.
Trimethylaminuria	Fish	Autosomal recessive resulting in low production of flavin containing monoxygenase enzyme 3 (FMO3).	Fish odor syndrome. Failure to breakdown trimethylamine, a build of which results in a fish odor.
Very long chain Acyl CoA dehydrogenase deficiency (LCAD)	Very long chain fatty acids	Autosomal recessive trait resulting from a mutation in the HADHA gene.	Prevents mitochondrial metabolism of very long chain fatty acids.

Other medically driven prohibitions include food allergies, the most common of which are to milk, egg, fish, crustacean shellfish, tree nuts, wheat, peanuts and soybeans which account for 90% of all food allergies in the US. The *Food Allergen Labeling and Consumer Protection Act of 2004* (FALCPA), effective January 1, 2006, requires labeling of any product containing these ingredients or a protein derived from one of these offending foods or incidental additives or flavors derived therefrom. Exceptions are limited to any highly refined oil derived from a major food allergen (e.g., peanut or soybean oil) or any food ingredient exempt from labeling under a *petition* or *notification process* specified in the law [14]. 

There are also a number of food-drug interactions, the consumption of one interfering with the metabolism of the other, which may result in an enhanced or abated effect of the drug (Table 2).

**Table 2:** Food drug interactions (used with permission from Kotsonis and Burdock [15]).

Enzyme or Transporter	Food	Drug
CYP1A2	Caffeine, theophylline, grapefruit juice (naringen and furanocourmarins bergmottin and dihydroxybergamotin), grape juice, cruciferous vegetables, apiaceous vegetables, cooked meat	Clozapine, fluvoxamine, imipramine
CYP2E1	Watercress and possibly other isothiocyanate-containing cruciferous vegetables; polyunsaturated fatty acids (corn oil, menhaden oil)	Ethanol, halothane, enflurane
CYP3A4	Grapefruit, orange juice, red wine, possibly other polyphenol-containing substances, St. Johns wort, garlic	Ketoconazole, cyclosporine, erythromycin, protease inhibitors, HMG-CoA reductase inhibitors
UGT and GST	Brussels sprouts, cabbage, watercress, broccoli	Acetaminophen, oxazepam, morphine, ibuprofen
P-glycopeptide and OATP	Vegetables, fruit juice, St. Johns wort	Digoxin, cyclosporine, parvastatin

UGT: uridine diphosphae glycuronosyltransferases; GST: glutathione-*S*-transferases; OATP: organic anion transporting polypeptides.

## 4. Toxin Incorporation during Growth, Storage or Processing

### 4.1. Environmental contaminants

#### 4.1.1. Selenium in grain

Selenium (Se) enters the food chain via plant and microorganism conversion of inorganic selenium to organically bound forms [16]. Selenium toxicity (*i.e.*, selenosis), caused by excessive selenium intake, has occurred on a large scale in seleniferous regions in China as the result of increased consumption of selenium-containing foods (approximate daily intake of 3–6.5 mg Se/day) [17]. The most common symptoms of selenosis are loss of hair, deformity, and loss of nails. Other reported symptoms include increased blood selenium levels, diarrhea, fatigue, a garlic-like odor of the breath and bodily secretions, irritability, peripheral neuropathy, and skin lesions [18]. Selenium intake levels that cause selenosis have not yet been well defined. Studies in China suggest that approximately 3–5 mg/day (0.05–0.08 mg/kg/day) will cause selenosis. Residents of seleniferous regions in South Dakota who consumed approximately 700 µg selenium/day (0.01 mg/kg/day) showed no symptoms of selenosis. The EPA has proposed an oral reference dose (RfD) of 0.005 mg/kg bw/day, or 350 µg/day [19].

#### 4.1.2. Methyl mercury in seafood

Exposure to elemental mercury is relatively rare, although was once an occupational disease of hat manufacturers as elemental mercury was used for the curing of animal pelts. Inhalation of the mercury fumes led to mental deterioration and subsequently named “mad hatter syndrome” [20].

Of interest to food toxicology, is the methyl derivative, methyl mercury, formed by bacterial action in an aquatic environment from anthropogenic and natural sources of elemental mercury. Anthropogenic sources include burning of coal (which contains mercury), chloralkali process and other sources of elemental mercury into aquatic environments. In the case of Minamata, Japan, there was a direct discharge of methyl mercury into the environment. Methyl mercury exposure may cause neurological paresthesias, ataxia, dysarthria, hearing defects and death. Developmental delays have been documented in children borne of mothers exposed to methyl mercury [21]. Other than direct exposure to methyl mercury, exposure usually comes about as the result of methyl mercury becoming incorporated into the food chain, moving up as each predator consumes the smaller and less fortunate animal. Near the peak of the food chain, methyl mercury becomes concentrated in fish including, bonito (*Sarda* spp.), halibut (*Hippoglossus* spp.), mackerel (*Scomberomorus* spp.), marlin (*Makaira* spp.), shark (all species), swordfish (*Xiphias gladius*), and bluefin tuna (*Thunnus* spp.). The selection of these species was based on historical data on levels of methyl mercury found in fish consumed in the U.S. The selection was also based on an FDA action level of 1.0 ppm in the edible portion of fish [22]. However, the allowable level of mercury depends on whether the mercury was “added”; that is, did the presence of mercury arise from an anthropogenic source (*i.e.*, was the fish caught in an area known for mercury discharge), or was not added and the result of mercury naturally present in the environment [23].

### 4.2. Naturally formed substances

#### 4.2.1. β-Thujone

Thujone, a monoterpene ketone, is the primary constituent of essential oils derived from a variety of plants, including sage (*Salvia officinalis*), clary (*Salvia sclarea*), tansy (*Tanacetum vulgare*), wormwood (*Artemisia* spp. and white cedar (*Thuja occidentalis* L.) [24]. Essential oils from these plants are used in herbal medicines, as flavorings in alcoholic drinks and fragrances throughout the world. Thujone is potentially toxic and the presence of alpha- or beta-thujone in food and beverages is regulated by law in several countries. In the US, thujone as an isolated substance is banned as an ingredient to be added to food and many of the natural thujone-containing plant oils (e.g., wormwood, white cedar, oak moss (*Evernia prunastri*) and tansy) are used as flavorings in food under the condition that the finished food is thujone-free [25]. Absinthe (made from wormwood) contains significant levels of thujone and is available in Spain, Denmark and Portugal. Wormwood itself is a popular flavoring for vodka in Sweden, while vermouth, chartreuse, and Benedictine all contain small levels of thujone [26]. Sage oil is used to provide the characteristic flavor in sausages, meats, condiments and sauces, and contains approximately 20–30% thujone (alpha- and beta-) [27,28]. Both alpha- and beta-thujone act as noncompetitive blockers of the gamma-aminobutyric acid (GABA)-gated chloride channel [29]. The essential oils of sage, hyssop (*Hyssopus officinalis* L.), and cedar all contain thujone and have been cited to have caused central nervous system effects characterized by tonic-clonic or solely clonic convulsions [30]. Thujone is believed to be the toxic agent in absinthism, a syndrome produced by the chronic use of absinthe, made from the essence of wormwood. The syndrome is characterized by addiction, hyperexcitability and hallucinations. The debilitating illnesses suffered by Vincent Van Gogh and Henri de Toulouse-Lautrec have been linked to absinthism, while the toxicity of thujone was a major factor in banning absinthe in the early 1900s [31]. A published case report detailed a male subject that drank about 10 mL of essential oil of wormwood (believing it was absinthe) and became agitated, incoherent and disoriented, subsequently developing renal failure [32]. The no observable effect limit (NOEL) for convulsions in subchronic toxicity studies in female rats was 5 mg/kg bw/day [24]. Detoxification of thujone is thought to occur via CYP450-dependent oxidation and subsequent glucuronidation and excretion [33]. The FDA limits exposure to β-thujone from *Artemisia* spp., when used as a natural flavoring substance or natural substance used in conjunction with flavors (21 CFR 182.20). 

#### 4.2.2. Prussic acid in cherry, apple and peach pits

Prussic acid (also known as hydrocyanic acid, hydrogen cyanide, or cyanide) is formed when cyanogenic glycosides found in leaves, cherry, apple and peach pits, oak moss and other plant tissues are damaged and come into contact with *beta*-glycosidase or emulsion enzymes. The enzymes release the cyanide from the glycoside, and the cyanide prevents the body’s cells from utilizing oxygen, resulting in cellular necrosis and tissue damage. The mucous membranes and blood are bright red as they are oxygenated, but the cells in the tissues cannot utilize the oxygen. Clinical signs of prussic acid poisoning include rapid breathing, trembling, incoordination and in extreme cases, respiratory and/or cardiac arrest [34]. Many fruit trees contain prussic acid glycosides in the leaves and seeds, but only negligible levels are present in the fleshy parts of the fruit [35]. In the west African tropics, cassava is consumed as a dietary staple and inappropriate handling of the cassava prior to processing and consumption can result in a chronic form of cyanide poisoning termed “tropical ataxic neuropathy”, the result of demyelinization of the optic, auditory, and peripheral nerve tracts [36]. 

Prussic acid as found in flavoring ingredients is limited to 25 ppm in cherry pits (*Prunus avium* L. or *P. cerasus* L.), cherry laurel leaves (*Prunus laurocerasus* L.), elder tree leaves (*Sambucus nigra* L.), and peach leaves (*Prunus persica* (L.) Batsch) (21 CFR 172.510); although the extract of bitter almond (*Prunus amygdalus* Batsch, *Prunus armeniaca* L., or *Prunus persica* (L.) Batsch) must be prussic acid free (21 CFR 182.20). There are no FDA regulations or guidelines restricting the presence of prussic acid in apple seed (*Malus* spp.), probably because extracts of these seeds have no economic value as flavor ingredients.

#### 4.2.3. Hypericin in St. John’s wort

**Figure 1:**
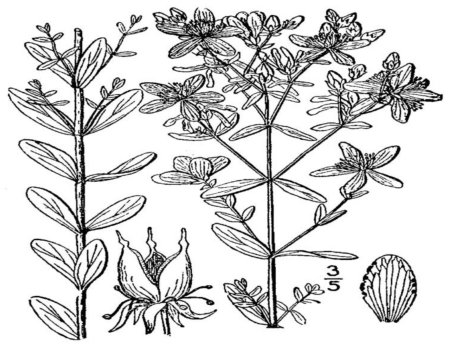
St. John’s wort (*Hypericum perforatum*) [40].

St. John’s wort (*Hypericum perforatum*; Figure 1) is an herbal thought to alleviate symptoms of depression, and standardized extracts of St. John’s wort are consumed typically in tablet or capsule form. The major active antidepressive constituents in St. John’s wort are thought to be hyperforin and hypericin [37,38]. The mechanism of action is not fully understood, but may involve inhibition of serotonin (5-HT) reuptake, similar to conventional antidepressive drugs. In this manner, hyperforin and hypericin taken in conjunction with other serotonin reuptake inhibitors may contribute to *serotonin syndrome*, a potentially life-threatening elevation of serotonin in the central nervous system. Hyperforin is also known to induce cytochrome P450 enzymes CYP3A4 and CYP2C9, which can lead to increased metabolism of certain drugs and decreased clinical response [39]. 

In large doses, St. John’s wort is poisonous to grazing animals, with published cases of livestock poisoning characterized by general restlessness and skin irritation, hindlimb weakness, panting, confusion, depression and in some instances, mania and hyperactivity resulting in the animal running in circles until exhausted [41]. In humans, consumption of St. John’s wort may result in photosensitization, and at high continuous doses, some liver damage may occur [39]. The FDA limits exposure to St. Johns wort (*Hypericum perforatum*), including the leaves, flowers, and caulis, by mandating that only hypericin-free alcohol distillate form may be used and then, only in alcoholic beverages (21 CFR 172.510).

#### 4.2.4. Goitrogens (glucosinolates) in Brassica spp.

Certain raw foods have been found to contain substances that suppress the function of the thyroid gland by interfering with the uptake of iodine, an essential nutrient in growth, cognitive function, and hormonal balance. A lack of functional iodine is known to result in cognitive deficiencies (e.g., Cretinism). The decrease in iodine uptake causes the thyroid gland to enlarge, forming a goiter. Foods that have been identified as goitrogenic include spinach, cassava, peanuts, soybeans, strawberries, sweet potatoes, peaches, pears, and vegetables in the *Brassica* genus, which include broccoli, brussels sprouts, cabbage, canola, cauliflower, mustard greens, radishes, and rapeseed [42]. Goiter has also been attributed to the consumption of large quantities of uncooked kale or cabbage. 

High temperatures (*i.e.*, cooking) inactivate the goitrogenic substances, collectively termed glucosinolates. Cassava (*Manihot esculenta*) is an essential dietary source of energy in the tropics, but contains high levels of linamarin, a glucosinolate. Cassava must be properly processed-dried, soaked in water or baked to effectively reduce the linamarin content [43]. Glucosinolates are sulfur-containing substances that are metabolized in the body by thioglucosidase to form thiocyanate, isothiocyanate, nitriles and sulfur. Under certain conditions the isothiocyanates undergo cyclization to form goitrins, increasing their potent goitrogenic activity. The oils from rapeseed (*Brassica napus*) must be analyzed for potential goitrins to circumvent potential goitrogenic activity when consuming these oils [44]. No FDA regulations were located for permissible concentrations of glucosinolates in human food. Glucosinolates (calculated as epi-progoitrin) and goitrin are limited to not more than 4% and 0.1% (respectively) of the seed meal of *Crambe abyssinica* (Crambe meal) obtained after the removal of the oil and used as an animal feed ingredient (21 CFR 573.310). 

#### 4.2.5. Erucic acid in rape

Rape (*Brassica napus* L. or *Brassica campestris* L.) is an annual herb of the mustard family native to Europe and is grown in the United States because it produces oil-rich seeds for cooking oil [45]. Rapeseed oil had been used for hundreds of years as oil for lamps and more recently as machine oil lubricant. Widespread use of rapeseed oil as a food ingredient was not considered until the late 1940s and 50s. However, early studies found that feeding high levels of rapeseed oil to rats significantly increased cholesterol levels in the adrenal glands and lipidosis in the cardiac tissue [46,47]. This effect was also noted in chickens, ducks and turkeys fed high levels of rapeseed oil, resulting in growth retardation, mortality, and a thickening of the epicardium and increased fibrous tissue in different areas of the myocardium [48]. Erucic acid was identified as the causative agent of these effects of rapeseed oil. Erucic acid is a long-chain fatty acid with one unsaturated carbon-carbon bond (C22:1). High levels of erucic acid have been liked to fatty deposit formation in heart muscle in animals [49]. Erucic acid is poorly oxidized by the mitochondrial β-oxidation system, especially by the myocardial cells, which results in an accumulation of erucic acid, producing myocardial lipidosis which has been reported to reduce the contractile force of the heart [50]. Although myocardial lipidosis due to erucic acid consumption has not been confirmed in humans, animal feeding studies confirmed the formation of myocardial lipidosis in a variety of animal species in a dose-dependent manner, which has been the standard assessment by government agencies of potential adverse effects in humans. Canola oil is obtained from Canola (Canadian oil, low acid), a rapeseed variety that was conventionally bred in the late 1970s in Canada to contain reduced levels of erucic acid and glucosinolates [51,52]. The FDA limits the amount of erucic acid in Canola oil to no more than 2% of the component fatty acids (21 CFR 184.1555).

#### 4.2.6. Furocoumarins

Furocoumarins represent a family of natural food constituents with phototoxic and photomutagenic properties. They are found mainly in plants belonging to the *Rutaceae* (e.g., citrus fruits) and *Umbelliferae* (e.g., parsnip, parsley, celery, carrots) families. Furocoumarins are produced in response to stress, to aid plants in defense against viruses, bacteria, fungi, insects and animals, and are regarded as natural pesticides [53]. Concentrations may also increase after exposure to UV radiation, changes in temperature, prolonged storage, or treatment with hypochlorite or copper sulfate (Chaudhary et al., as cited in Wagstaff 1991 [53], p. 270 and Beier *et al*., as cited in Ashwood-Smith [54], p. 916). 

The three most active furocoumarins in producing photodermatitis are psoralen, 5-methoxypsoralen (5-MOP, bergapten), and 8-methoxypsoralen (8-MOP, xanthotoxin or methoxsalen) [55]. In the presence of near UV light (320–380 nm), these three linear furocoumarins can form adducts with DNA and DNA-crosslinks. The consequences of these photoadditions to cells are cell death, mutations and chromosome aberrations [54]. In the presence of ultraviolet A radiation, 5-MOP and 8-MOP produce skin tumors in experimental animals. At a chronic dose of 37.5 mg/kg bw/day in the diet, 8-MOP produces increased incidences of tubular cell hyperplasia, adenomas, and adenocarcinomas of the kidney and carcinomas of the Zymbal gland in rats [56]. Cases of skin cancer have been reported in patients treated with 8-MOP and long-wave ultraviolet light for treatment of psoriasis or mycosis fungoides [57,58]. IARC has classified 5-MOP and 8-MOP plus ultraviolet radiation in group A (probably carcinogenic in humans) and in group 1 (carcinogenic to humans), respectively [57,59].

Citrus fruits, especially grapefruit, produce a variety of chemicals in their peels that may have adverse interactions with drugs. Typically, citrus fruit juice is produced utilizing the whole fruit, including the peel. One chemical found in the peel is bergamottin (also known as bergamot), a natural furanocoumarin that is known to inhibit some isoforms of the cytochrome P450 enzyme (CYP) 3A4 [60]. Inhibition of this enzyme prevents oxidative metabolism of certain drugs, resulting in an elevated concentration of a drug in the bloodstream [61]. Bergamot and other chemicals in citrus (e.g., lime, grapefruit, orange, lemon) oils [62] are also phototoxic, causing significant toxicity to the skin when exposed to sunlight [63]. 5-Methoxypsoralen, the most phototoxic constituent of bergamot oil, showed mutagenic activity in bacterial assays and clastogenic effects in mammalian cells in culture when exposed to UV light [64]. 

Celery reportedly contains 100 ppb psoralens (100 micrograms/kg) and parsnips as much as 40 ppm (40 mg/kg) [65]. The estimated dietary intake of furocoumarins for people eating furocoumarin-containing foods (est. 80% of the population) is 1.31 mg/day [53], which is approximately 0.022 mg/kg bw/day for a 60 kg human. This is approximately 1000-fold lower than the 13-week dietary no observable adverse effect level (NOAEL) for liver toxicity in the rat (25 mg 8-MOP/kg bw/day) and 1700-fold lower than the dietary dose that has been shown to induce cancer in rats (37.5 mg/kg). Therefore, the risk of developing liver toxicity or cancer due to ingestion of psoralens in the diet is low.

In humans, the phototoxic threshold dose of furocoumarin mixtures after dietary exposure is of the order of 10 mg 8-MOP plus 10 mg 5-MOP, which is equivalent to about 15 mg 8-MOP per person. This phototoxic threshold dose is not reached by the consumption of celery roots and other conventional vegetables under normal dietary habits, which result in intake of approximately 2–8 mg furocoumarins per person [66]. Therefore, ordinarily dietary exposure to psoralens is not considered to be a significant risk for development of photodermatitis, albeit the margin of safety is low [65]. There are no FDA regulations or guidelines specific to the presence of furocoumarins in food.

#### 4.2.7. Amylase inhibitors

Naturally occurring inhibitors of α-amylase are found in aqueous extracts of wheat, rye and kidney beans. The physiological role of α-amylase inhibitors in plants is not well understood, but may protect them against insect infestation. In mammals, some amylase inhibitors have been shown to attenuate the normal increase in blood glucose that occurs after ingestion of starch. However, since α-amylase inhibitors have been shown to be inactivated by gastric acid, pepsin or pancreatic proteinases, their potential as “starch blockers” is limited [67]. α-Amylase inhibitors were once added to foods as “starch blockers” to limit carbohydrate absorption for the purpose of weight loss; however, the FDA later determined that at least this use of α-amylase inhibitors was as drug, and they were consequently taken off the market [68]. 

α-Amylase inhibitor protein is a major allergen (referred to as Asp o 2) that has been implicated in the development of occupational toxicity known as “baker’s asthma disease” [69]. Although α-amylase inhibitor protein is naturally found in wheat flour, it is also found in flour in which α-amylase from *Aspergillus oryzae* has been added to enhance carbohydrate fermentation by yeast [70]. Consequently, α-amylase inhibitor protein can be potentially found in baked products that are derived from sources other than wheat. Cases of food allergy have been reported in people ingesting bread containing α-amylase inhibitor protein. Symptoms of allergy include sneezing, rhinorrhea, oropharyngeal itching, hoarseness, cough and dyspnea [71].

High α-amylase inhibitor activity against human salivary α-amylase has been found in wheat flour (590 units/g), whole wheat flour (351 units/g) and whole rye flour (186 units/g). Bread baking reduces the activity by 80–100%, depending on type. The activity in uncooked spaghetti (248 units/g) is reduced more than 98% by 15 minutes of boiling. Boiling of red beans for 1.5 hours reduces activity to undetectable levels [71]. However, α-amylase has been shown to retain some allergenic activity when heated to 200 °C (Baur *et al.*, as cited in Phadia AB 2010 [72], p. 2). 

#### 4.2.8. Lectins in legumes

Lectins are a group of glycoproteins that are present in high levels in legumes (e.g., black beans, soybeans, lima beans, kidney beans and lentils) and grain products [73,74]. Lectins can reversibly bind to carbohydrates without altering their covalent structure [73]. The ability of lectins to bind to and agglutinate red blood cells is well known and used for blood typing—hence the lectins are commonly called hemagglutinins. Lectins also can bind avidly to mucosal cells and interfere with nutrient absorption from the intestine [75]. Because the ability of the lectins to cause intestinal malabsorption is dependent on the presence of enteric bacteria, it has been hypothesized that lectins may also produce toxicity by facilitating bacterial growth in the GI tract [76].

Lectins isolated from black beans can produce growth retardation when fed to rats at 0.5% of the diet, and lectin from kidney beans causes death within two weeks when fed to rats at 0.5% of the diet. Soybean lectin produces growth retardation when fed to rats at 1% of the diet. The castor bean lectin ricin (one of the most toxic natural substances known) is notorious for causing deaths of children, and has been used as an instrument of bioterrorism [75].

Phytohaemagglutinin (PHA) is a lectin found in significant quantities (as much as 2.4–5% of total protein) in legumes such as red or white kidney beans, green beans and fava beans. PHA has a number of different properties, including the ability to induce mitosis, affect membrane transport and permeability to proteins, and agglutinate red blood cells. Rats fed a diet containing 6% PHA exhibit weight loss, associated with malabsorption of lipid, nitrogen and vitamin B12 [76]. PHA from red kidney beans inhibits sodium and chloride absorption in the rabbit ileum, indicating that PHA can affect electrolyte transport in the gut [77]. Symptoms of toxicity to PHA in humans such as nausea, vomiting, or diarrhea occur within three hours of ingestion. Recovery generally occurs within four or five hours of onset [78].

There are no FDA regulations or guidelines restricting the presence of lectins in food, but the FDA does provide recommended cooking practices prior to consuming legumes. Concentrations of PHA (and other lectins) are higher in uncooked than cooked beans. A raw, red kidney bean can contain up to 70,000 hemagluttinating units (hau). Most lectins are reduced by moist, but not dry heat. Therefore, steaming or boiling causes a significant reduction in concentrations of lectins in beans. Boiling for at least ten minutes has been shown to reduce hau in beans by 200-fold. Because cooking temperatures under 176 °F do not destroy lectin, use of slow cooking and/or a crockpot is not advised for cooking beans [79]. 

#### 4.2.9. Anti-thiamine compounds

Substances that act on the availability of vitamins are commonly referred to as antivitamins. These include materials that can cause a deficiency of vitamins by competing with vitamins in various metabolic reactions as the result of similar chemical structure or destroying or decreasing the effects of a vitamin by modifying the molecular conformation or by forming a complex [67]. 

Thiaminase cleaves thiamine (vitamin B1) at the methylene linkage, rendering it biologically inactive. Activity of thiaminase requires a cosubstrate—usually an amine or sulfhydryl-containing protein such as proline or cysteine. Thiaminase is found in fish, crab, clams and in some fruits and vegetables such as blueberries, black currants, red beets, Brussels sprouts and red cabbage [67].

Thiamine is an essential vitamin involved in energy production. Thiamine deficiency is associated with impaired pyruvate utilization, resulting in a shortage of cellular ATP. In humans, thiamine deficiency may lead to weakness and weight loss. Severe thiamine deficiency produces “beri-beri”, a disease characterized by anorexia, cardiac enlargement, and muscular weakness leading to ataxia [80]. Cooking destroys thiaminases in fish and other sources. There are no FDA regulations or guidelines specific to the presence of thiaminase in food. 

#### 4.2.10. Pyrrolizidine alkaloids

**Figure 2:**
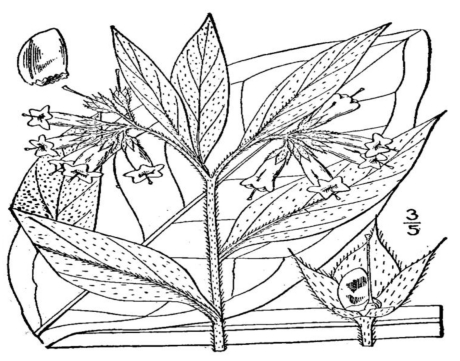
Comfrey (*Symphytum officianale* L.) [83].

Pyrrolizidine alkaloids (PAs) are found in some plants of the Apocyanacae, Asteraceae, Boraginaceae, Compositae (*Senecionae* and *Eupatoriae*), Fabaceae, Leguminosae (*Crotalaria*), Rannuculaceae and Scrophulariaceae families. Herbs such as comfrey root and leaf (*Symphytum* spp.) (Figure 2), coltsfoot leaf and flower (*Tussilago farfara*) and borage leaf (*Borago officinale*), and several species of *Eupatorium* typically contain high levels of PAs. Humans are exposed to PAs through the accidental contamination of foodstuffs and intentional ingestion of PA-containing vegetables and herbal medicines. Serious incidences of illness have been reported in people consuming cereal grains that are contaminated with the seeds of PA-containing plants [81]. PAs are also present in milk from cows and goats and in honey [82].

The pyrrolizidine structure is based on two fused, five-membered rings that share a bridgehead nitrogen atom, forming a tertiary alkaloid. The rings contain a hydroxymethylene group at the C-1 position and a hydroxyl group at the C-7 position, forming a necine base. Several PAs that contain unsaturated necine rings are hepatotoxic, mutagenic, teratogenic and/or carcinogenic. Toxicity is thought to be due to enzymatic conversion of PAs to pyrroles, which act as alkylating agents [67]. Pyrroles formed in the liver can travel to the lungs, causing thickening of the pulmonary vasculature and pulmonary hypertension [82].

The sale of comfrey products for internal use has been banned in the United States and Canada [82]. However, comfrey tea is still widely available. It is estimated that consumers of comfrey tea could be ingesting up to 5 mg of PAs per day (Speijers and Egmond, as cited in Deshpande 2002b [81], p. 368), or 0.083 mg/kg bw/day. The range of toxic doses in humans is thought to be 0.1–10 mg/kg per day [84], although the World Health Organization has reported a case of veno-occlusive disease in a subject ingesting 0.015 mg PAs/kg of body weight per day from comfrey.

#### 4.2.11. Rhubarb and oxalic acid

Oxalic acid (oxalate) is generally found in rhubarb (0.2–1.3%), tea (0.3–2.0%), spinach (0.3–1.3%), parsley (1.7%) and purslane (1.3%), but may also be found in asparagus, broccoli, Brussels sprouts, collards, lettuce, celery, cabbage, cauliflower, turnips, beets, peas, coffee, cocoa, beans, potatoes, berries, and carrots [67,73,85].

Oxalic acid is an organic acid that can bind calcium and other minerals, making them insoluble and decreasing their bioavailability. Ingestion of foods containing high concentrations of oxalates may cause decreased bone growth, kidney stones, renal toxicity, vomiting, diarrhea, convulsions, coma and impaired blood clotting [73]. The significant role oxalate plays in kidney stone development is exemplified by the fact that approximately 65% of kidney stones consist of calcium oxalate [86].

Using the oral LD_50_ value of 375 mg/kg in rats, it has been estimated that ingestion of approximately 22 g of oxalic acid could be lethal to a 59 kg human [85]. Because approximately 4.5 kg of rhubarb leaves would have to be ingested in order to achieve a lethal dose, it has been hypothesized that documented cases of fatal rhubarb poisoning in humans were due to consumption of some other substance than oxalic acid [67]. 

Because cooking does not remove oxalate, and mineral complexes with oxalate are insoluble in water, oxalates are somewhat difficult to remove from foods. Therefore, diets rich in oxalate-containing foods should be supplemented with minerals such as calcium or potassium to prevent deficiencies. Limits on oxalic acid have been cited in ferric ammonium ferrocyanide and ferric ferrocyanide when used as color additives (21 CFR 73.1298 and 21 CFR 73.1299) with oxalic acid or its salts at not more than 0.1% of the colorant. 

#### 4.2.12. Zucchini and cucurbitacins

Members of the *Cucurbitacea* family (zucchini, cucumbers, pumpkins, squash, melons and gourds) produce cucurbitacins (oxygenated tetracyclic terpenes) that act as movement arresters and compulsive feeding stimulants for Diabriticine beetles (corn rootworms and cucumber beetles). Cucurbitacins are among the most bitter compounds known, and in nanogram quantities they deter most non-Diabrotic herbivores [87]. 

Because cucurbitacins act as feeding stimulants, they are added to insecticidal baits to increase efficacy [88]. Therefore, dietary exposure to cucurbitacins could occur through ingesting plants that normally contain them or by ingesting plants to which cucurbitacin-containing pesticides have been applied. 

Under normal circumstances, cucubitacins are produced at low enough concentrations that are not perceived as being bitter by humans. In response to stresses such as high temperatures, drought, low soil fertility and low soil pH, concentrations in fruits such as cucumbers may increase and cause the fruits to have a bitter taste [89]. Occasional cases of stomach cramps and diarrhea have occurred in people ingesting bitter zucchini. Twenty–two cases of human poisoning from ingestion of as little as 3 grams of bitter zucchini were reported in Australia from 1981 to 1982, and in Alabama and California in 1984. The cultivar implicated in the Australia poisonings was “Blackjack” [90]. There are no FDA regulations or guidelines specific to the presence of cucurbitacins in food. 

#### 4.2.13. Coumarins (tonka bean, woodruff, clover)

Coumarin (2H-1-benzopyran-2-one) is found in herb teas made from tonka beans (*Dipteryx odorata*), melilot (*Melilotus officinalis* or *Melilotus arvensis*) and woodruff (*Asperula odorata*), the flavoring oil of bergamot (from *Citrus bergamia*) and the spice cassia (*Cinnamomum cassia*; sometimes sold as cinnamon) [91]. Coumarin is liberated from the glycoside melilotoside (an ether of glucose bonded with an ester bond to coumarin) on drying coumarin-containing herb material. 

Molds present in spoiled sweet (Melilotus) clover and other hay products can metabolize coumarin to dicoumarol, which is similar in structure to vitamin K [92]. Vitamin K is necessary to activate prothrombin, which is converted to the blood clotting substance thrombin. By inhibiting vitamin K, dicoumarol promotes bleeding. Concentrations of dicoumarol in fodder >10 ppm have been responsible for fatalities by hemorrhaging in cattle [91]. 

The addition of coumarin to food in the United States was banned in 1954, based on reports of hepatoxicity in rats. However, because a number of foods contain coumarin, humans ingest approximately 0.02 mg coumarin/kg bw/day. The chronic administration of high doses of coumarin causes liver tumors in the rat and liver and lung tumors in the mouse. Overall, available data indicate that coumarin is not genotoxic. It is thought that the carcinogenicity of coumarin is caused by metabolism to toxic epoxides. Because doses of coumarin that cause toxicity and carcinogenicity in the lung and liver of experimental animals are more than 100 times the maximum human intake, exposure to coumarin from food poses no health risk to humans [93]. 

The addition of coumarin is prohibited in 21 CFR 189.130. The regulation notes that coumarin is found in tonka beans and extract of tonka beans, among other natural sources, and is also synthesized. It has been used as a flavoring compound, therefore addressing not just natural products (which would include buffalo grass or sweetgrass (*Hierochloe odorata*) used in flavoring vodka and other natural sources (see above)), as well as synthesized coumarin. Further, according to the regulation, “(b) Food containing any added coumarin as such or as a constituent of tonka beans or tonka extract is deemed to be adulterated under the act, based upon an order published in the Federal Register of March 5, 1954 (19 Federal Register 1239).” An analytical method for detection of coumarin in foods is specified in 21 CFR 189.130.

#### 4.2.14. Phytates and phytic acid

Phytic acid (also referred to as phytate) is found in bran and germ of many plant seeds and in grains, legumes and nuts. Phytic acid is a simple sugar (myo-inositol) containing six phosphate sidechains, and as such, is a dietary source of phosphorus and an effective chelator of divalent cations such as zinc, copper, iron, magnesium and calcium [67,94]. Studies indicate that phytate-mineral complexes are insoluble in the intestinal tract, reducing mineral bioavailability [73]. Phytate also has been shown to inhibit digestive enzymes such as trypsin, pepsin, α-amylase and ß-glucosidase. Therefore, ingestion of foods containing high amounts of phytate could theoretically cause mineral deficiencies or decreased protein and starch digestibility. Vegetarians that consume large amounts of tofu and bean curd are particularly at risk of mineral deficiencies due to phytate consumption.

Because phytate-rich foods are digested at a slower rate and produce lower blood glucose responses than foods that do not contain phytate, it has been hypothesized that phytate could have a therapeutic role in management of diabetes [67]. It also may have utility as an antioxidant [95]. However, because the beneficial effects of phytate are outweighed by its ability to cause essential mineral deficiencies, consumption of a diet containing high amounts of phytate is not recommended. Food manufacturers are developing methods to reduce phytate in foods, such as addition of the microbial phytase, which releases phosphates from the inositol backbone of phytate [96].

Phytate is fairly heat stable, but can be removed by soaking or fermentation [67]. The soybean has one of the highest phytate levels of any grain or legume, and requires a long period of fermentation for reduction [94]. In people who consume large amounts of soy products, mineral deficiencies can be prevented by consumption of meat or dairy products or use of supplemental vitamins. There are no FDA regulations or guidelines restricting the presence of phytates in food.

#### 4.2.15. Hypoglycin in Ackee

**Figure 3:**
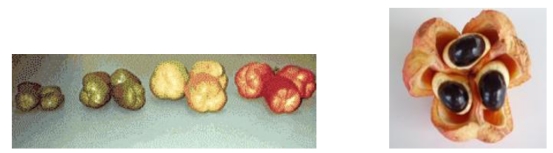
Unripe Ackee Fruit (left panel) and ripe Ackee Fruit (right panel) [100].

Ackee (*Blighia sapida*; Figure 3) is the national fruit of Jamaica and is also found in other Caribbean nations, Central America, South American and southern Florida [97]. Consumers of the unripe fruit sometimes suffer from “Jamaican vomiting sickness syndrome” allegedly caused by the alkaloids hypoglycin A (HGA) and B. Levels of HGA in the opened, ripe fruit are undetectable, making opened fruit safe for consumption [98]. 

The hypoglycin toxin (L-methylenecyclopropylalanine) inactivates several flavoprotein acyl-CoA dehydrogenases, causing disturbances of the oxidation of fatty acids and amino acids [99]. This leads to a secondary inhibition of gluconeogenesis which can precipitate an extreme, dangerous drop in blood-glucose levels (hypoglycemia) that can be fatal. Symptoms of poisoning from unripe ackee fruit occur within 6 to 48 hours of ingestion and include drowsiness, repeated vomiting, thirst, delirium, fever or loose bowels. Exhaustion of the muscular and nervous systems, collapse, coma, and death may ensue [100,101].

Dietary exposure to hypoglycin in Jamaicans ranges from 1.21–89.28 micrograms/gram ackee [102]. Ingestion of one 100 gram fruit could therefore result in a dose of approximately 300 micrograms/kg bw in a 30 kg child. This dose is approximately one-fifth of the maximum tolerated dose of HGA in male and female rats of 1500 micrograms/kg bw/day [103], indicating that normal use levels of ackee do not have a large margin of safety.

The importation of canned ackee fruit into the United States is restricted to certain manufacturers to insure that only properly ripened ackees are used for canning [104], and the FDA routinely analyzes incoming shipments of ackee for hypoglycin levels that could be a health concern, having issued a recall of canned ackee fruit for this very reason in 2005. If hypoglycin poisoning is expected, glucose, fluids and electrolytes should be administered. Antiemetics may be used to control vomiting and benzodiazepines to control seizures. Endotracheal intubation should be performed in people exhibiting seizures or coma [97].

#### 4.2.16. Safrole

Safrole (1-allyl-3,4-methylenedioxybenzene) is found in aromatic oils of nutmeg (*Myristica fragrans*), cinnamon (*Cinnamomum verum*) and camphor (*Cinnamomum camphora*) and is a major constituent of oil of sassafras (*Sassafras albidum*) [105]. Prior to being banned as a food additive in the United States in 1960, safrole was commonly used to flavor root beer and other foods. Most commercial “sassafras teas” and root beers are now artificially flavored as a result of the FDA ban (21 CFR 189.180). 

At a concentration of 1% in the diet, safrole produces weight loss, testicular atrophy, bone marrow depletion and malignant liver tumors in rats [106]. Based on sufficient evidence of carcinogenicity in experimental animals, safrole is reasonably anticipated to be a human carcinogen [107]. The mechanism of carcinogenicity is thought to involve cytochrome P450 catalyzed hydroxylation of safrole to 1’-hydroxysafrole, and its subsequent metabolism to highly reactive electrophiles that bind to DNA [108]. 

Despite the FDA ban, sassafras is still a popular ingredient in herb teas and preparations [73]. The hazardous dose of sassafras oil for humans (which typically contains 80% safrole) is considered to be 0.66 mg/kg [109]. This may be exceeded by ingesting sassafras tea, which has been estimated by Segelman and Bisset (as cited in Burfield 2009 [109], p. 3) to give a dose of 3 mg/kg for a 60 kg individual. 

#### 4.2.17. Myristicin

**Figure 4:**
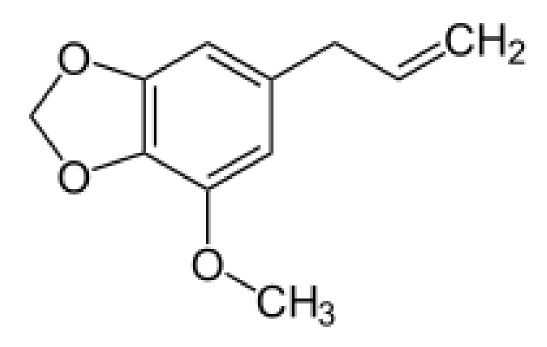
Structure of myristicin.

Myristicin (Figure 4) is a naturally occurring insecticide and acaracide that is found in nutmeg and mace (*Myristica* spp.) at concentrations of 1.3% and 2.7%, respectively [110]. It is also present in black pepper, carrot, celery parsley and dill [67]. It is estimated that the average total intake of myristicin from dietary sources is “in the order of a few mg per person per day” [110].

Myristicin is a weak inhibitor of monoamine oxidase, and is structurally related to mescaline. At a dose level of 6–7 mg/kg bw, it may cause psychotropic effects in man, such as increased alertness, and a feeling of irresponsibility, freedom and euphoria. Unpleasant symptoms, such as nausea, tremor, tachycardia, anxiety and fear have also been reported in humans ingesting this dose. Although the metabolism of myristicin resembles that of safrole, there is no evidence to suggest that myristicin is carcinogenic [110]. There are no FDA regulations or guidelines specific to the presence of myristicin in food.

At the concentrations normally present in spices or food, the likelihood of toxicity arising from myristicin is low. However, ingestion of greater than 5 grams of nutmeg (corresponding to 1–2 mg/kg bw myristicin) has produced toxicological symptoms in humans that are similar to alcohol intoxication. Because the myristicin content of nutmeg is approximately 1–3%, it is likely that components of nutmeg in addition to myristicin contribute to nutmeg toxicity [110].

#### 4.2.18. Tomatine in tomatoes

The leaves, stems and unripe fruit of the tomato plant contain α-tomatine, a steroidal alkaloid containing D-xylose, D-galactose, and two molecules of D-glucose. Tomatine is toxic to a number of different fungi, thereby acting as a natural fungicide. It has been hypothesized that the toxic effects of tomatine on fungi are due to the ability of tomatine to complex with membrane sterols, causing membrane disruption [111]. 

Currently, there is no evidence to suggest that tomatine is a substance of concern. There are no reports of acute toxicity in humans due to ingestion of green tomatoes and there are no FDA regulations or guidelines specific to the presence of tomatine in food. Ingestion of a rare variety of ripe tomato (*Lycopersicon esculentum* var. cerasiforme) that contains up to 5 mg tomatine/g of dry weight has no adverse effects on natives who commonly ingest them [112]. 

Concentrations of tomatine decrease as tomatoes ripen, and ripe fruit contains approximately 36 mg per a 100 gram tomato [73]. Microwaving or frying does not reduce content of tomatine, and delayed-ripening varieties of tomatoes contain similar concentrations of tomatine as other tomatoes [113]. At this time, there is no evidence to suggest that a diet high in green tomatoes would be injurious to human health. Tomatine forms strong, insoluble complexes with cholesterol *in vitro*, and has been shown to lower plasma LDL cholesterol in hamsters [114], suggesting that it may have beneficial effects on blood lipids of humans.

#### 4.2.19. Japanese star anise

Chinese star anise (*Illicium verum*) is a common source of anethole, a popular flavoring ingredient. On the other hand, Japanese star anise (*Illicium anisatum*) is scientifically recognized as highly poisonous and not fit for human consumption. Japanese star anise contains the potent neurotoxins anisatin and neoanisatin, as well as the neurotoxic sesquiterpene lactone veranisatins that are normally found in other kinds of star anise, including Chinese star anise [115]. 

Brewed “teas” containing star anise have been associated with illnesses affecting about 40 individuals, including approximately 15 infants. The illnesses ranged from serious neurological effects, such as seizures, to vomiting, jitteriness and rapid eye movement. Due to the potential for adulteration, on September 10, 2003, the FDA issued an advisory to the public not to consume “teas” brewed from star anise, until the FDA is able to differentiate between the Japanese star anise and Chinese star anise, which does not contain anisatin [116]. 

### 4.3. Substances formed as the result of product abuse

#### 4.3.1. Glycoalkaloids (solanine and chaconine) in potatoes

The glycoalkaloids α-solanine and α-chaconine are natural pesticides that are produced in potatoes. α-Solanine is also found in eggplant, apples, bell peppers, cherries, sugar beets and tomatoes [74,117]. The only difference between α-solanine and α-chaconine is the sugars in the trisaccharide potion of the molecule, *i.e.*, glucose with two rhamnoses for α-solanine and a glucose, galactose and a rhamnose for α-chaconine [118].

Depending on variety and storage conditions, concentrations of α-chaconine and α-solanine in potato tubers vary between 0.5–635 ppm (0.0005–0.64 mg/g potato) and 5–125,100 molecule ppm (0.005–25.1 mg/g potato), respectively (Beckstrom-Sternberg, as cited in Tice 1998 [117], p. 9). Although glycoalkaloids are found throughout the potato tuber, the greatest concentrations are in the sprouts, peels and sun-greened areas [74]. The FDA considers the maximum acceptable glycoalkaloid content to be 20–25 mg/100 g fresh potato weight (or 200–250 ppm) (Crocco, as cited in FDA 2008 [119], p.1). Under current FDA regulations, 20 milligrams of solanine per 100 grams (a small potato) can render it unfit to eat. 

Synthesis of α-chaconine and α-solanine is stimulated by light, mechanical injury, aging and potato beetle infestation [117,120]. Exposure of potatoes to light in the field or marketplace can lead to glycoalkaloid concentrations that are unsafe for human consumption. Concentrations of solanine in green or blighted potatoes have been shown to increase by seven fold [73]. 

The symptoms of acute toxicity to α-solanine and α-chaconine are due to their ability to act as inhibitors of acetylcholinesterase and disruptors of cell membranes. Glycoalkaloid doses of 1 to 5 mg/kg have been shown to be acutely toxic to humans, and doses of 3 to 6 mg/kg have resulted in death [117]. Symptoms of glycoalkaloid toxicity in humans include drowsiness, itchiness in the neck region, increased sensitivity (hyperesthesia), labored breathing and gastrointestinal symptoms (abdominal pain, nausea, vomiting and diarrhea) [74]. 

α-Solanine and α-chaconine are not mutagenic or only weakly mutagenic *in vitro*, are not genotoxic *in vivo*, and are embryotoxic and teratogenic to experimental animals. Teratogenic effects in mammals include central nervous system abnormalities (e.g., exencephaly, cranial bleb, encephalocele, and anophthalmia), mild hydronephrosis, hydroureter, and irregular or fused ribs. Although one human case study reported a correlation between the severity of potato late-blight and the incidence of spina bifida, no other studies in humans have found a correlation between the consumption of potatoes and birth defects [117]. There is no evidence that α-solanine and α-chaconine are carcinogenic in animals or humans.

In 1993, the National Institute of Environmental Health Sciences determined that the average consumption of glycoalkaloids from potatoes was 12.75 mg glycoalkaloids/person/day (0.18 mg/kg bw based on a bw of 70 kg) [117], which is approximately one-fifth of the lowest dose that has been shown to produce acute toxicity in humans (1 mg/kg bw). 

#### 4.3.2. Furocoumarin in parsnips

Ceska *et al*. reported that older 'spoiled' and diseased parsnips freely available in grocery stores may contain furocoumarin concentrations 2500% higher than fresh parsnips [121]. Microbial infection of parsnip roots can result in a dramatic increase in furocoumarin levels. Furocoumarin concentrations (the sum of five furocoumarins: angelicin, isopimpinellin, 5-MOP, 8-MOP and psoralen) in freshly harvested parsnips are generally lower than 2.5 mg/kg and do not increase after storage at −18 °C for up to 50 days. In contrast, storage of whole parsnips (but not cubes or homogenate) at 4 °C resulted in a marked biphasic increase of furocoumarin concentrations (to approximately 40 mg/kg) after seven or 38 days of storage. A dramatic increase in furocoumarin concentrations (up to 566 mg/kg) was observed when whole parsnips were kept at room temperature over 53 days, resulting in a visible microbial (mold) infection [122].

In celery, infection with fungal pathogens has been shown to produce timethylpsoralen (which is absent from plants that are not infected) and increased concentrations of 8-MOP. The resulting “pink rot” has caused repeated outbreaks of photophytodermatitis in commercial celery handlers [55]. Fungal infection also has been shown to stimulate a 155-fold increase in furocoumarin production by carrots (Ceska *et al*., as cited in Wagstaff 1991 [53], p. 268). There are no FDA regulations or guidelines specific to the presence of furocoumarins in food.

### 4.4. Substances formed as the result of processing

#### 4.4.1. Heterocyclic aromatic amines

There are two major classes of heterocyclic aromatic amines (HAAs). Pyrolytic HAAs are formed from the pyrolysis of amino acids or proteins at high temperature and aminoimidazoarenes (AIAs) are formed from creatine, free amino acids and monosaccharides, via the Maillard reaction. HAAs are present in many protein-rich foods of animal origin including cooked meat, fish, poultry and gravies and sauces derived from pan residues and scrapings of cooked meats. The formation and yield of HAAs are dependent on cooking temperature and time (concentrations increase with higher temperatures and longer cooking times), cooking technique and equipment (concentrations of HAAs in meat are generally higher after grilling and panfrying than broiling or roasting), and the ability of HAA precursors to migrate to the surface [123].

The AIAs 2-amino-3-methylimidazo-[4,5-f]quinoline (IQ), 2-amino-3,4-dimethylimidazo[4,5-f]quinoline (MeIQ) and 2-amino-3,8,dimethylimidazo[4,5-f]quinoxaline (MeIQx) are among the most potent mutagens ever tested in the Ames assay. The pyroltic AIA 2-amino-1-methyl-6-phenylimidazol(4,5-b)pyridine (PhIP) and the HAAs 2-amino-1,4-dimethyl-5H-pyrido[4,3-b]indole (Trp-P-1), 2-amino-4-methyl-5H-pyrido[4,3-b]indole (Trp-P-2), 2-amino-9H-pyrido[2,3-b]indole (AαC), 2-amino-3-methyl-9H-pyrido[2,3-b]indole (MeAαC) are also mutagenic. PhIP accounts for 75% of the mass of genotoxic material that has been attributed to HAAs in fried ground beef. Therefore, the potential for genotoxicity due to PhIP may be higher than that of more genotoxic HAAs in meat consumers [123]. 

Several HAAs are carcinogenic in rodents after long-term dietary administration. The doses required to induce tumors at a 50% rate (TD_50_) vary for each HAA, and range from 0.1 to 64.6 mg/kg bw/day [123]. Four HAAs (IQ, MeIQ, MeIQx and PhIP) are “reasonably anticipated to be human carcinogens” [124]. Due to the fact that exposure to HAAs in cooked meats is highly variable (concentrations in cooked meat may range from <1 to 500 ng/g), it has been estimated that the risk of developing cancer from exposure to HAAs in food is anywhere from 50 in one million to one in a thousand [123]. Currently, no tolerable upper limit of exposure to HAAs has been established. 

#### 4.4.2. Polycyclic aromatic hydrocarbons

Polycyclic aromatic hydrocarbons (PAHs) are known carcinogens that are formed from the incomplete combustion of fossil fuels such as wood, coal and oil. PAHs can enter the food chain from environmental contamination or from food processing. Foods containing the highest concentrations of PAHs include cooked or smoked meat or fish, smoked or cured cheese, tea and roasted coffee. Grilling or broiling of meat, fish or other foods over intense heat or direct contact with flames promotes production of PAHs. In general, concentrations of PAHs in meat are highest after charcoal grilling, followed by smoking, roasting and steaming. Concentrations of PAHs in smoked foods are influenced by temperature, type of wood, oxygen concentration and type of smoker. Concentrations of PAHs in tea dried over burning wood, oil or coal are generally higher than in tea dried over air, and coffee beans that are roasted over a direct fire contain higher concentrations than beans that do not come in contact with flames [125]. 

The European Commission’s (EC) Scientific Committee on Food and the Joint FAO/WHO Expert Committee on Food Additives (JECFA) has concluded that thirteen different PAHs are genotoxic and carcinogenic benz[a]anthracene, benzo[b]fluoranthene, benzo[j]fluoranthene, benzo[k]fluoranthene, benzo[a]pyrene, chrysene, dibenz[a,h]anthracene, dibenzo[a,e]pyrene, dibenzo[a,h]pyrene, dibenzo[a,i]pyrene, dibenzo[a,l]pyrene, indeno[1,2,3-cd]pyrene and 5-methylchrysene. Three of the four PAHs that have been tested for carcinogenicity in rats after oral exposure (benz[a]anthracene, benzo[a]pyrene and dibenz[a,h]anthracene) are carcinogenic. The estimated high and safe levels of intake of the benchmark PAH benzo[a]pyrene are 0.01 and 100 μg benzo[a]pyrene/kg bw/day, respectively, indicating that the estimated intake of PAHs in food is 10,000-fold lower than the level that is expected to cause toxicity in humans [126]. Currently, no tolerable upper limit of exposure to PAHs has been established by the FDA.

#### 4.4.3. Acrylamide

**Figure 5:**
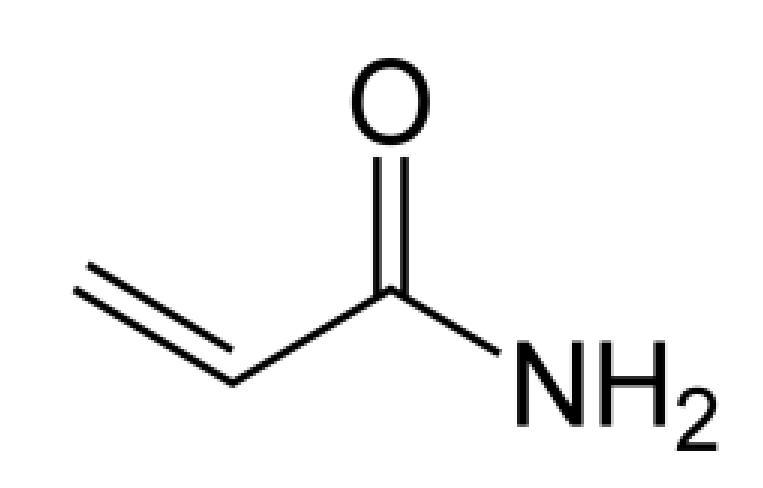
Structure of acrylamide.

Acrylamide (Figure 5) is found in a number of starch-based foods that are fried or baked at temperatures greater than 120 °C (248 °F), including bread, bakery products, breakfast cereal, and potato products (e.g., chips, french fries) [127]. It also is found in cocoa-based products and coffee. Acrylamide is formed via a Maillard reaction, a reaction between the carbonyl group of a reducing sugar and the nucleophilic group of an amino acid. Although a number of carbohydrates can be used as the source of the carbonyl group, the amino acid required for the formation of acrylamide is asparagine. 

Acrylamide is mutagenic and has been shown to be a neurotoxicant, reproductive toxicant and carcinogen in experimental animals and is classified by IARC as a probable human carcinogen. The main metabolite, glycidamide (an epoxide) is thought to be responsible for genotoxicity [127]. In humans, the only toxicological effect that has been linked to acrylamide is neurotoxicity in individuals occupationally exposed to high levels. Epidemiological studies have failed to show an increased risk of cancer from either occupational or dietary exposure to acrylamide and reproductive toxicity has not been reported in humans exposed to acrylamide [128]. Acrylamide is a unique substance that exemplifies the concept that the structure of the substance greatly influences the toxicity, as acrylamide is an animal feed ingredient (thickener and suspending agent) only when a part of a long-chain polymer having a minimum molecular weight of 3 million and a viscosity range of 3,000 to 6,000 centipoises at 77 °F. The residual acrylamide cannot be more than 0.05% (21 CFR 573.120). 

In 2005, JECFA estimated that average and high intake consumers ingest 1 or 4 μg/kg bw/day acrylamide from food, respectively. Using a NOAEL for neurotoxicity of 200 μg/kg bw/day in animals, margins of safety of 200 and 50 for the average and high intake groups were derived, respectively. Utilizing a benchmark dose of 0.3 mg/kg bw/day and a NOAEL of 2 mg/kg bw/day for development of mammary tumors or reproductive in rats (respectively), higher margins of safety were calculated for carcinogenicity (300 and 75, respectively) and reproductive toxicity (200 and 50, respectively) [128].

Exposure to acrylamide can be reduced by avoiding deep-fried foods, soaking potato slices before cooking, cooking french fries at lower temperatures and to a lighter color, and toasting bread to a lighter color [127].

#### 4.4.4. Chloropropanols

Chloropropanols are formed in hydrolyzed vegetable proteins (HVP) produced by hydrochloric acid (HCl) hydrolysis of proteinaceous by-products from edible oil extraction, such as soybean meal, rapeseed meal and maize gluten [129,130]. The chloropropanol most commonly found in food is 3-MCPD (3-monochloropropane-1,2-diol), although others may also be present, including 2-MCPD (2-monochloropropane-1,3-diol), 1,3-DCP (1,3-dichloro-2-propanol), and 2,3-DCP (2,3-dichloro-1-propanol) [130]. The two most widely studied chloropropanols are 3-MCPD and 1,3-DCP. It is thought that 3-MCPD is formed as a result of a reaction between a source of chlorine (chlorinated water or sodium chloride) in a food or a food contact material and a lipid. Two basic pathways have been proposed: thermally driven and enzyme-catalyzed (generally lipase) reactions. Direct precursors are thought to be glycerol and chloride. Recent work has also suggested glycidol (2,3-epoxy-1-propanol) as a precursor. 1,3-DCP is thought to arise from 3-MCPD.

High concentrations of 3-MCPD have been found in acid hydrolyzed HVP (acid-HVP), and soy or oyster sauce produced using an acid hydrolysis process. Other foods that may contain 3-MCPD are cereal, toasted bread, coffee, cheese, licorice, baked goods, processed garlic, liquid smokes, malts, cured or smoked meat or fish or foods containing acid-HVP as a savory ingredient (soups, prepared meals, savory snacks, gravy mixes and stick cubes [129,130,131,132]. Foods containing 1,3-DCP include raw meat and soy sauce produced using an acid hydrolysis process [129].

In rats and mice, 3-MCPD is toxic to the kidney, producing renal tubule hyperplasia. It is also carcinogenic in rats when given in high doses over prolonged periods. Although 3-MCPD is genotoxic *in vitro*, it is not *in vivo*. The UK Committee on Carcinogenicity of Chemicals in Food, Consumer Products and the Environment (COC) has concluded that 3-MCPD is unlikely to present a carcinogenic risk to man, provided the exposure is 1000 times lower than the no observed effect level (NOEL) of 1.1 mg/kg bw/day for tumorigenicity. JECFA set a tolerable daily intake (TDI) of 2 μg 3-MCPD/kg of body weight in 2001 and a maximum allowable content of free 3-MCPD in liquid condiments at 0.4 mg/kg (400 μg/kg) in 2008 [130]. Assuming 400 μg/kg 3-MCPD is present in soy sauce, a 60 kg human would have to ingest 300 g of soy sauce (approximately two-thirds of a 444 mL bottle) per day to achieve the TDI. The FDA has provided a policy statement stating that acid-H[V]P or Asian sauces that contain 3-MCPD at levels greater than 1 ppm are not Generally Recognized As Safe (GRAS); therefore, these ingredients are unapproved food additives [133]. 

1,3-DCP is hepatotoxic, genotoxic and induces a variety of different types of tumors in rats. Therefore, 1,3-DCP is considered to be a potential carcinogen in humans. In 1993, FAO/WHO and JECFA concluded in that 1,3-DCP is an undesirable contaminant in food and that levels should be reduced to as low as “technologically achievable” [131]. 

#### 4.4.5. Furan

**Figure 6:**
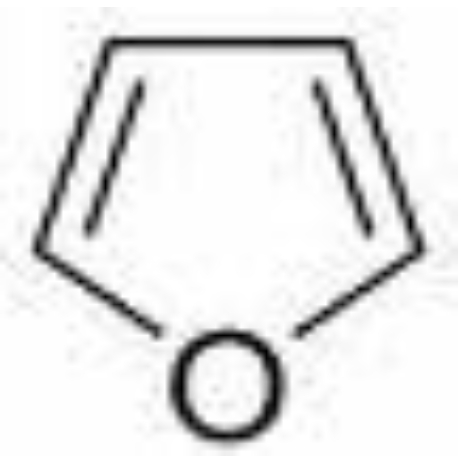
Structure of Furan.

Furan (Figure 6) is a by-product of high-energy and thermal treatment of carbohydrate. Meat and vegetable containing foods that are heat processed in cans and jars (such as soups, pastas, sauces, gravy and baby food) and brewed coffee, typically contain the highest concentrations. Concentrations of furan present in food and coffee range from undetectable to approximately 175 μg/kg [134]. Coffee powders may contain up to 5000 μg/kg on a dry weight basis. Although the mechanism of formation of furan in food is not completely understood, it can be synthesized from vitamin C, amino acids, reducing sugars, organic acids, carotenes and polyunsaturated fatty acids in the presence of heat [135]. 

Furan is mutagenic and clastogenic in a number of *in vitro* mammalian cell assays, causes damage to chromosomes in mice, and is carcinogenic in both rats and mice after oral administration [134,136,137,138]. Furan is classified by IARC as possibly carcinogenic to humans [134].

In the United States and Europe, exposure to furan from food is estimated to be a maximum of 1.00 and 1.75 μg/kg bw/day, respectively [134]. The upper estimate of consumption is approximately 300 and 1000-fold lower than the NOAELs for cytotoxicity and hepatocarcinogenicity of 500 and 2000 μg/kg bw in female B6C3F1 mice, determined by Moser *et al.* [136]. 

Mitigation of furan in foods is difficult because the mechanism for its formation in food is unclear. Due to the fact that furan is volatile, it is thought that concentrations can be reduced by heating food in open containers or leaving ready-to-eat foods open to air after preparation. However, the effectiveness of this strategy in reducing exposure to furan has yet to be demonstrated [135]. Currently, there are no FDA regulations specific to the level of furan in food. 

#### 4.4.6. Trans fatty acids

Trans fatty acids (also known as trans fat) are the sum of all unsaturated fatty acids that contain one or more isolated double bonds in a trans configuration. Trans fatty acids more closely resemble saturated fatty acids than cis unsaturated fatty acids because their trans configuration makes them rigid. Trans fatty acids in the diet originate from two sources. The first is from bacterial hydrogenation in the forestomach of ruminants, which produces trans fatty acids that are found in beef and mutton fat, milk and butter. Trans fatty acids are also produced from the hydrogenation of liquid oils (mainly of vegetable origin). This produces solid fats and partially hydrogenated oils such as margarines, spreads, shortenings and frying oil, which are more stable than liquid oils [139].

Biochemically, trans-fatty acids act similarly to saturated fatty acids, raising low density lipoprotein (LDL) cholesterol and decreasing high-density lipoprotein (HDL) cholesterol levels [139]. High intakes of trans fatty acids have been associated with an increased risk of coronary heart disease (CHD) independent of other risk factors in large epidemiological studies [140]. A tolerable upper limit of trans fatty acids has not been set because any incremental increase in the intake of trans fatty acids increases the risk of coronary heart disease [141].

In the US, the main sources of intake of trans fatty acids are baked goods (28%), fried foods (25%), margarine, spreads and shortenings (25%), savory snacks (10%), milk and butter (9%) [139]. In 1996, processed foods and oils accounted for 80% of the trans fat in the diet [141]. In 1999, the FDA estimated that the average daily intake of trans fat in the United States is about 5.8 grams or 2.6% of calories per day [142]. It has been hypothesized that replacing 2% energy from trans fatty acids with 2% energy from oleic acid would reduce mean plasma LDL cholesterol concentration by 0.08 mmol/L, and increase plasma HDL concentration by 0.08 mmol/L. These changes could reduce the incidence of CHD by 5–15% [139].

Due to increased efforts by food manufacturers to reduce or eliminate the use of partially hydrogenated vegetable fat in food production, it is estimated that trans fatty acid content of processed foods has decreased over the last decade [143]. 

#### 4.4.7. Nitrosamines formed during drying, curing and preserving

Nitrosamines are formed from the interaction of nitrites or other nitrosating agents with amines in food (or *in vivo*), under acidic conditions. Nitrites may be directly added to food or can be formed from bacterial reduction of nitrate. Nitrites and nitrates may occur naturally in water or foods such as leafy vegetables due to the use of fertilizer, or may be added to foods to prevent growth of *Clostridium botulinum*, or to add color or flavor [144]. 

Nitrosamines have been found in a variety of different foods such as cheese, soybean oil, canned fruit, meat products, cured or smoked meats, fish and fish products, spices used for meat curing, and beer and other alcoholic beverages [145]. Beer, meat products and fish are considered the main sources of exposure. Drying, kilning, salting, smoking or curing promotes formation of nitrosamines [146].

The nitrosamines most frequently found in food are nitrosodimethylamine (NDMA), *N*-nitrosopyrrolidine (NPYR), *N*-nitrosopiperidine (NPIP), and *N*-nitrosothiazolidine (NTHZ) [146]. NDMA, NPYR, NPIP are reasonably anticipated to be human carcinogens based on evidence of carcinogenicity in experimental animals [145,147,148]. Evidence from case-control studies supports an association between nitrosamine intake with gastric cancer, but not esophageal cancer in humans [149]. 

Levels of nitrosamines have been declining during the past three decades, concurrent with a lowering of the nitrite used in food, use of inhibitors such as ascorbic acid and use of lower operating temperatures and indirect heating during food processing. Based on an estimated exposure level of 3.3–5.0 ng/kg bw/day, the and the benchmark lower limit of 60 μg/kg bw/day, a margin of error associated with a low level of concern (12,000–18,2000) has been derived for NDMA, the most common nitrosamine in food [146]. 

Although current FDA regulations do not limit nitrosamine levels in foods, the FDA has provided an action level of 10 ppb for individual nitrosamines in both consumer and hospital rubber baby bottle nipples, while the FDA limits the approval of nitrites in curing mixes to the FDA-regulated food additive process (21 CFR 170.60), with the approval of sodium nitrite as a food additive (food preservative) (21 CFR 172.175). The USDA monitors finished meat products to insure that nitrite is not present in amounts exceeding 200 ppm (9 CFR 424.21). 

#### 4.4.8. Biogenic amines

Biogenic amines are normally formed in humans by normal cellular metabolism. In food, biogenic amines are mainly formed from microbial decarboxylation of amino acids. They are commonly found in fermented meat, beverages and dairy products, sauerkraut, and spoiled fish. The main biogenic amines in food are histamine, tyramine cadaverine, putrescine, spermidine and spermine. The two biogenic amines that have been associated with acute toxicity are histamine and tyramine. Putresine, spermine, sperimidine and cadaverine are not toxic in and of themselves, but may react with nitrite or nitrate to form nitrosamines (see Section 4.4.7 above) [150]. 

Scombrotoxicosis is a common seafood-borne disease associated with the consumption of toxic levels of histamine in spoiled scombroid fish such as tuna (*Thunnus* spp.), mackerel (*Scomber* spp.), saury (*Cololabis* saira) and bonito (*Sarda* spp.). Red wine may also contain relatively high levels of histamine. Symptoms of histamine intoxication from food are similar to allergies to other substances and include sneezing, nose congestion, breathing difficulties and urticaria [150]. 

Consumption of tyramine may precipitate migraine headache or a hypertensive crisis. The most serious case reports of tyramine toxicity have occurred in people consuming aged cheese. Because monoamine oxidase inhibitor (MAOI) drugs inhibit metabolism of amines, people taking these drugs may be particularly susceptible to tyramine toxicity. Whereas 200–800 mg of dietary tyramine induces only a mild rise in blood pressure in unmedicated adults, 10–25 mg may produce a serious adverse event in those taking MAOI drugs. Other potentiating factors for tyramine toxicity include alcohol consumption, gastrointestinal distress and exposure to other amines [150].

Efforts taken by food manufacturers to reduce biogenic amine concentrations in fermented foods include using amine-negative starter cultures, adding probiotic bacterial strains alone or in combination with starter cultures, high pressure processing or low-dose gamma radiation [150]. FDA guidelines specify 50 mg/100 g as the toxic concentration of histamine in scombroid fish and the agency has published guidance on how to control levels [151]. 

## 5. Substances Passed from Animals to Humans

### 5.1. Toxins in seafood

#### 5.1.1. Toxins involving algae

Consumption of seafood contaminated with algal toxins results in five different syndromes, paralytic, neurotoxic, amnesic, or diarrhetic shellfish poisoning and ciguatera fish poisoning [152].

##### 5.1.1.1. Paralytic shellfish poisoning

Paralytic shellfish poisoning (PSP) is caused by the consumption of molluscan shellfish contaminated with heterocyclic guanidines called saxitoxins. Currently, over 21 known saxitoxins are produced by dinoflagellate species from three genera: *Alexandrium*, *Gymnodium* and *Pyrodinium*. Toxicity is caused by binding of saxitoxins to voltage-dependent sodium channels, which blocks neuronal activity. The primary site of action in humans is the peripheral nervous system. Symptoms of toxicity include tingling and numbness of the perioral area and extremities, loss of motor control, drowsiness, and incoherence. Ingestion of 1–4 mg saxitoxin has resulted in death from respiratory paralysis [152]. 

Outbreaks of PSP have occurred worldwide, due to the fact that saxitoxin-producing species of dinoflagellates can live in either temperate or tropical waters. Saxitoxins are not inactivated by cooking, and must be mitigated at their source to prevent ingestion. PSP is prevented by large-scale, proactive monitoring programs and rapid closures of harvest in areas containing dinoflagellate algal blooms [153]. In the United States, the permissible level of saxitoxin equivalents in shellfish is 80 micrograms/100 grams [154].

##### 5.1.1.2. Neurotoxic shellfish poisoning

The dinoflagellate *Karenia brevis* produces brevetoxins that are lethal to fish, but not to mollusks such as oysters, clams and mussels. Consequently, they can accumulate in healthy-appearing mollusks to concentrations that are toxic to humans who ingest them. *Karenia brevis* brevitoxins cause the syndrome known as neurotoxic shellfish poisoning (NSP), which affects sodium transport in the autonomic nervous system and causes inhibition of neuromuscular transmission in skeletal muscle. 

NSP is usually a relatively mild illness and should not be confused with the more serious condition of PSP. NSP symptoms usually occur within three hours of ingesting contaminated shellfish and may include abdominal pain, nausea and vomiting, vertigo, malaise, generalized muscle weakness, ataxia, incoordination, chills, headache, myalgia, a reversal of hot/cold temperature sensation and progressive parasthesias. Dilated pupils, bradycardia and convulsions may occur in cases of severe poisoning [155]. Unlike PSP, no deaths have been reported from NSP [152]. 

*K. brevis* is the organism that is usually responsible for the red tides in the Gulf of Mexico and along the southern Atlantic coast of North America. Blooms along the west coast of Florida occur regularly [156]. Biotoxin control plans that are implemented during period of red tide are generally effective in preventing NSP, but have not eliminated NSP entirely. 

The FDA has established an action level of 0.8 ppm (20 mouse units/100 g) brevetoxin-2 equivalents [154].

##### 5.1.1.3. Amnesic shellfish poisoning (Domoic acid)

**Figure 7:**
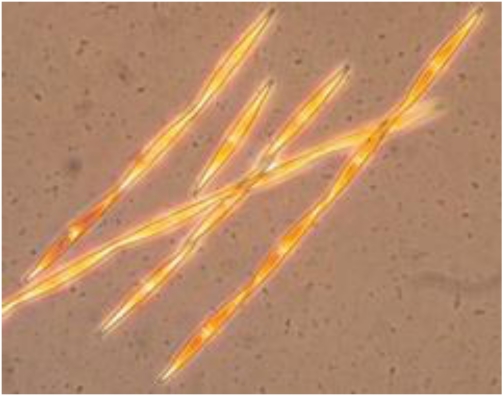
*Pseudo*-*nitzchia* [157].

Amnesic shellfish poisoning (ASP) is caused by domoic acid produced by diatoms of the genus *Pseudo*-*nitzchia* (Figure 7), which are consumed by mussels, scallops, clams and crabs. Domoic acid is a water-soluble, tricarboxylic amino acid that is a structural analog of the neurotransmitter glutamate and is a glutamate receptor agonist. Persistent activation of the kainite glutamate receptor causes an increase in intracellular calcium, which can cause neuronal cell death and lesions of the brain where glutamineric pathways are concentrated. Areas of the brain involved in learning and memory processing are particularly susceptible [152]. The symptoms of ASP are gastroenteritis, dizziness, disorientation, lethargy, seizures and loss of short term memory. Respiratory difficulty, coma and death may ensue [153]. Human toxicity has occurred after ingestion of 1–5 mg/kg domoic acid [152].

In 1987, approximately 100 people became ill and died in Prince Edward Island, Canada, after eating contaminated mussels. In 1991, domoic acid poisoning caused the deaths of numerous pelicans and cormorants in Monterey Bay that ingested sardines and anchovies. Domoic acid also was responsible for a massive sea lion kill in Monterey Bay in 1998 [158]. *Pseudo*-*nitzchia* and domoic acid are now closely monitored throughout the world [159]. The FDA has established an action level of 20 ppm for domoic acid, except in the viscera of Dungeness crab, where 30 ppm is permitted [154]. Regulatory guidance has been effective in preventing ASP in humans, since no human outbreaks of ASP have occurred since 1987.

##### 5.1.1.4. Diarrhetic shellfish poisoning

Diarrhetic shellfish poisoning (DSP) is caused by the production of okadaic acid and dinophysistoxins in the dinoflagellates *Dinophysis fortii* or *Prorocentrum lima*, which are consumed by mollusks. Okadaic acid and dinophysistoxins are inhibitors of serine/threonine phosphatases, critical components of signaling cascades that regulate a number of cellular processes involved in metabolism, ion balance, neurotransmission and cell cycle regulation [152].

Compared to other types of shellfish poisoning, symptoms of DSP are relatively mild, and generally consist of diarrhea, abdominal cramps, nausea, chills or vomiting within 30 minutes to a few hours after consumption of DSP toxins. Symptoms generally resolve within 2–3 days, with or without medical treatment [153]. Diarrhea is most likely due to the hyperphosphorylation of proteins (including ion channels) in the intestinal epithelia, resulting in impaired water balance and fluid loss. The long term consequences of low level exposure to DSP toxins may be more serious, as they have been shown to be tumor promoters [152]. The FDA has established an action level of 0.2 ppm okadaic acid plus 35-methyl okadaic acid (DXT 1) [154].

##### 5.1.1.5. Ciguatera poisoning

Ciguatera fish poisoning (CFP) is caused by the dinoflagellate *Gambierdiscus toxicus*, which grows on filamentous macroalgae associated with coral reefs. The lipophilic precursors to ciguatoxin are biotransformed to ciguatoxins in herbivorous fish and invertebrates that consume the macroalgae, and bioaccumulate in large carnivorous fishes associated with coral reefs. High ciguatoxin concentrations may be found in barracuda, snapper, grouper and jacks [152]. 

Ciguatoxins are structurally related to the brevetoxins and compete with brevetoxin for binding to the same site on the voltage-dependent sodium channel. However, because ciguatoxin has a higher binding affinity for the site than brevetoxin, the toxic potency of ciguatoxin is higher than that of brevetoxin. The threshold level for toxicity in humans is estimated to be 0.5 ng/g [152]. 

CFP is estimated to affect over 50,000 people worldwide each year. The symptoms of CFP generally include gastrointestinal disturbances (nausea, vomiting and diarrhea) within 2–6 hours, followed by neurologic symptoms such as numbness of the perioral area and extremities, a reversal of hot/cold temperature sensation, muscle and joint aches, headache, itching, tachycardia, hypertension, blurred vision and paralysis. In rare cases, CFP is fatal [152].

Inasmuch as ciguatoxin is produced by organisms that live beneath the surface and is not routinely monitored for concentration in seafood, the only way to prevent consumption is to completely abstain from ingesting tropical reef fish, as the occurrence of toxic fish is sporadic, and not all fish of a given species or from a given locality will be toxic [153]. Currently, there are no FDA regulations limiting levels of ciguatoxins in fish, although a recent publication suggests an advisory level of 0.1 ppb pacific ciguatoxin equivalent (P-CTX-1) toxicity values in fish from the tropical Atlantic, Gulf of Mexico, Caribbean, and 0.01 ppb P-CTX-1 equivalent toxicity in fish from Pacific regions [160].

#### 5.1.2. Toxins not involving algae

##### 5.1.2.1. Gempylotoxin

**Figure 8:**
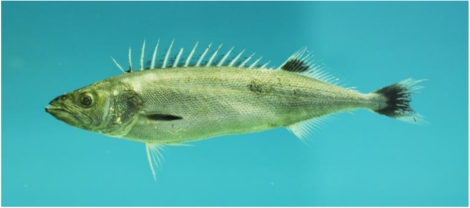
Juvenile Oilfish (*Ruvettus* pretiosus) [165].

There are naturally occurring toxins in some species that do not involve marine algae. Escolar (*Lepidocybium flavobrunneum*, Figure 8), and Oilfish or Cocco (*Ruvettus pretiosus*), a marine fish of the snake mackerel family, are sometimes sold under the category of “butterfish”, and contain a strong purgative oil, that when consumed can cause diarrhea known as Gempylid Fish Poisoning, Gempylotoxism or Keriorrhea [161]. The toxin consists of wax esters (C32, C34, C36 and C38 fatty acid esters), the primary component of which is C_34_H_66_O_2_[162]; these constitute a substantive portion of the lipid present in these fish (14–25% by weight). Escolar oil contains >90% wax esters [163]. Ingestion of fish containing wax esters in large amounts, coupled with their indigestibility and low melting point, results in diarrhea [164]. No tolerances have been established, and the FDA recommends avoidance of these fish [161]. 

##### 5.1.2.2. Tetramine in whelks

Tetramine is a toxin found in the salivary glands of *Buccinum*, *Busycon* or *Neptunia* spp., a type of whelk or sea snail that is distributed in temperate and tropic waters and has long been a food source for humans. Whelk are associated with a heat-stable neurotoxin, tetramine, which upon ingestion produces, among other symptoms, eyeball pain, headache, dizziness, abdominal pain, ataxia, tingling in the fingers, nausea and diarrhea [166,167]. Power *et al*. report that the highest concentration of tetramine is in the salivary gland (up to 6530 μg/g), but varies according to season [168]. Reid *et al.* reported levels of 37.5 μg tetramine/g of salivary gland tissue [166]. Because the whelk is a predator of bivalves, it is assumed the toxin is used for food procurement [168]. Although the FDA recommends removal of the salivary gland to avoid possible intoxication [154], tetramine is present in other tissues, albeit at lesser concentrations [169].

##### 5.1.2.3. Trimethylamine oxide

The meat of the Greenland shark (*Somniosus microcephalus*) and the related member of the dogfish family, the pacific sleeper shark (*Somniosus pacificus*), is known to be poisonous to both man and dogs. The causative agent is trimethylamine oxide, which breaks down to trimethylamine in the gut, probably by enteric bacteria. The result is absorption of trimethylamine, which acts as a neurotoxin, producing ataxia in both man and dogs. However, the flesh may be consumed if boiled several times with changes of water, or as the Inuit prepares it, by burying it in the ground and allowing the meat to go through several freezing and thawing cycles [170,171,172].

### 5.2. Toxins from animal, non-seafood sources passed on to humans

#### 5.2.1. Grayanotoxins in honey and direct contact with food

**Figure 9:**
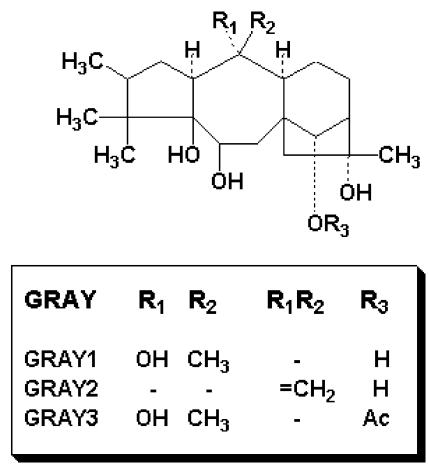
Grayanotoxins [173].

Rhododendrons and azaleas (*Rhododendron* spp.), oleander (*Nerium oleander* or *Nerium indicum*), mountain laurel (*Kalmia latifolia*) and sheep laurel (*Kalmia angustifolia*), all produce grayanotoxins (Figure 9) whose action is to bind to sodium channels in muscle, including the heart. Although not all rhododendrons produce grayanotoxins (also known as oleander toxin, andromedotoxin, acetylandromedol or rhodotoxin), several species growing in the US are known to produce grayanotoxins and include *Rhododendron occidentale*, *Rhododendron macrophyllum* and *Rhododendron albiflorum*, all in the western US. Grayanotoxin is also found in the eastern US, within the botanical family Ericaceae, to which rhododendrons belong and are probably the most important sources of the toxin [173]. 

Grayanotoxin consists of a series of cardiac glycosides: thevetin, convallarin, steroidal, helleborein, ouabain, and digitoxin. At first, sympathetic nerves are paralyzed; the cardiotoxin stimulates the heart muscles similar to the action of digitalis, and gastric distress ensues. Symptoms start out as nausea, vomiting, abdominal pain and diarrhea; followed by tremor, drowsiness and ataxia. In severe cases, ectopic beats occur which may be followed by ventricular tachycardia and fibrillation. The origin of toxicity may be honey (made from the nectar of the flowers), milk from a cow having eaten the foliage and meat (e.g., hot dogs) roasted on oleander sticks [15,174]. The pooling of large quantities of grayanotoxin-containing honey or milk during commercial processing typically dilutes grayanotoxin to nontoxic levels. There are no FDA regulations specific to grayanotoxin levels in foods.

#### 5.2.2. Tremetol contamination of milk from white snakeroot

“Milk sickness” also known as “puking fever”, “sick stomach”, “the slows” and “the trembles”, was a mysterious scourge of the Midwest United States in the 18th and 19th centuries. Thousands of people have been reported as dying, including Abraham Lincoln’s mother, Nancy Hanks Lincoln. In humans, milk sickness is characterized by loss of appetite, listlessness, weakness, vague pains, muscle stiffness, vomiting, abdominal discomfort, constipation, foul breath and finally, coma. For many years the origin of milk sickness was unknown, because there was nothing comparable in Europe (origin of most of the pioneers) and the outbreaks were sporadic. It was not recognized until the late 19th and early 20th century, that white snakeroot (*Ageratina altissima* née *Eupatorium rugosum*) and rayless goldenrod (*Bigelowia* spp., *Haplopappus heterophyllus* and *Isocoma pluriflora*) when eaten by cattle, was the source. The sporadic nature of outbreaks became clear when it was realized that cattle would consume these plants in over-grazed pasture or in years of drought; additionally, the toxin levels in plants can vary considerably, making identification of the source of poisonings difficult. Tremetol or tremetone is the toxic agent and consists of a mixture of sterols and derivatives of methyl ketone benzofuran. The three major benzofuran ketones are tremetone, dehydrotremetone and 3-oxyangeloyl-tremetone [173,174,175,176,177]. Currently, there is no USDA guidance specific to tremetol levels in dairy products. 

## 6. Conclusions

Given the state of the science, the pressure on the food supply and the development of new products, the FDA has performed admirably in protecting the consumer from exposure to toxins in food with its judicious use of warning labels, action levels, tolerances, specifications, prohibitions and the ability conferred by Congress to declare substances “unsafe” or “unfit for food.” However, the FDA cannot protect consumers absolutely from exposure to toxins normally present in foods. At normal levels of food consumption, there is little potential for toxicity from natural food toxins. Nevertheless, there is always the possibility of an idiosyncratic response or undetected contamination. 
